# Paclitaxel Chemotherapy Elicits Widespread Brain Anisotropy Changes in a Comprehensive Mouse Model of Breast Cancer Survivorship: Evidence From *In Vivo* Diffusion Weighted Imaging

**DOI:** 10.3389/fonc.2022.798704

**Published:** 2022-03-23

**Authors:** Lauren D. Otto, Kathryn L. G. Russart, Praveen Kulkarni, Dana M. McTigue, Craig F. Ferris, Leah M. Pyter

**Affiliations:** ^1^ Institute for Behavioral Medicine Research, Ohio State University Wexner Medical Center, Columbus, OH, United States; ^2^ Arthur G. James Comprehensive Cancer Center and Solove Research Institute, Ohio State University, Columbus, OH, United States; ^3^ Center for Translational Neuroimaging, Department of Psychology and Pharmaceutical Sciences, Northeastern University, Boston, MA, United States; ^4^ Department of Neuroscience, Ohio State University, Columbus, OH, United States; ^5^ Department of Psychiatry and Behavioral Health, Ohio State University, Columbus, OH, United States

**Keywords:** survivor, DTI/DWI, fatigue, translational, mammary tumor, cytokines, comorbidities

## Abstract

Breast cancer is one of the most common diseases in the United States with 1 in 8 women developing the disease in her lifetime. Women who develop breast cancer are often post-menopausal and undergo a complex sequence of treatments including surgery, chemotherapy, and aromatase inhibitor therapy. Both independently and through potential interactions, these factors and treatments are associated with behavioral comorbidities reported in patients (e.g., fatigue), although the underlying neurobiological mechanisms are poorly understood. Currently, brain imaging is the most feasible way to assess neurobiology in patients. Indeed, breast cancer patients display alterations in white matter connections and chemotherapy is associated with decreased white and gray matter in the corpus callosum and cortex as well as decreased hippocampal volume. However, imaging in breast cancer rodent models is lacking, impeding translation of the mechanistic neurobiological findings made possible through modeling. Furthermore, current rodent models of breast cancer often lack the complexity of typical multimodal breast cancer treatments, thereby limiting translational value. The present study aimed to develop a comprehensive model of post-menopausal breast cancer survival using immunocompetent ovariectomized mice, including an orthotopic syngeneic tumor, surgical tumor removal, chemotherapy, and aromatase inhibitor therapy. Using this model, we systematically investigated the cumulative effects of chemotherapy and hormone replacement therapy on neurostructure and behavior using diffusion weighted imaging, open field test, and spontaneous alternation test. Our previous findings, in a simplified chemotherapy-only model, indicate that this regimen of chemotherapy causes circulating and central inflammation concurrent with reduced locomotor activity. The current study, in the more comprehensive model, has recapitulated the peripheral inflammation coincident with reduced locomotor activity as well as demonstrated that chemotherapy also drives widespread changes in brain anisotropy. Validating the clinical relevance of this comprehensive rodent breast cancer model will allow for additional neurobiological investigations of the interactions among various cancer components associated with behavioral comorbidities, as well as the relationship between these mechanisms and neurostructural imaging changes that can be measured in cancer patients.

## Introduction

Over 3.8 million women in the United States are breast cancer survivors, with more than 280,000 new diagnoses predicted for 2021 (1). With advances in treatment and screening, 90% of these patients survive at least 5 years (1). However, 17-98% of patients and survivors report negative behavioral side effects before, during, and after treatment, including fatigue, mood disorders, and cognitive impairments (2–4). Fatigue is one of the most common behavioral comorbidities in breast cancer patients (5) and is often reported after chemotherapy treatment but can also occur even before chemotherapy, suggesting additive causal roles of stress, tumor biology, and surgery (6). Fatigue can persist years after treatment ends (7–9). Even mild behavioral consequences undeniably reduce quality-of-life, which in turn reduces work performance and employability (10, 11), increases medical costs (12), and decreases treatment adherence (13–15).

The central mechanisms of cancer-associated fatigue are not yet elucidated, but fatigue after chemotherapy treatment is associated with altered brain microstructure (16, 17). For example, fatigued breast cancer survivors display dynamic differences in white matter connections between specific regions of the brain (18). Furthermore, chemotherapy treatment corresponds with reduced white and gray matter in the corpus callosum and cortex (19) and reduced hippocampal volume (20). In some cases, these structural changes persist over 20 years post-chemotherapy (21) and may be progressive (22). However, these effects vary with chemotherapeutic agent and regimen and radiotherapy treatment (23, 24). Microstructural damage analyzed by diffusion tensor imaging (DTI) has also been reported with fatigue in non-oncological human diseases (25, 26).

In addition to structural brain changes, chemotherapy, as well as surgery and tumor biology, causes systemic inflammation (27, 28). Indeed, circulating proinflammatory markers in chemotherapy patients (e.g., c-reactive protein and interleukin [IL]-6) positively correlate with behavioral comorbidities including fatigue (29–33). Using rodent models, both tumors and chemotherapy independently cause behavioral abnormalities and increases in circulating and neuroinflammatory markers (34). Indeed, peripheral inflammatory signals from a tumor or from cell death caused by chemotherapy treatment can propagate into the brain and result in local neuroinflammation that alters neuronal functions and behavior (33). Systemic and neuroinflammation have also been implicated in structural changes in white and gray matter (35).

Current research on the neurobiological mechanisms underlying these breast cancer behavioral comorbidities has limitations as many rodent models are lacking critical components of the typical breast cancer paradigm: syngeneic, orthotopic, estrogen receptor positive (ER+) tumors (often no tumors), post-menopausal reproductive status (many studies in males), tumor resection surgery, repeated chemotherapy cycles, and various other consecutive treatments (e.g., aromatase inhibitors). As most breast cancer patients are post-menopausal, have ER+ tumor status, and receive anti-estrogen therapy, the inclusion of these aspects in a model of breast cancer enhances validity, particularly given the known role of estrogen in mood, cognition, and brain structure (36–39). Combining imaging techniques, such as diffusion weighted imaging (DWI), with neurobiological analyses in comprehensive rodent breast cancer models will improve the current translatability of mechanistic research findings. Our goal for this project was to create a comprehensive breast cancer mouse model that incorporates multiple clinically relevant factors that could influence the brain to more accurately represent the breast cancer patient and treatment experience and to understand their combined effects using a translational neuroimaging technique. Our extensive model of a typical post-menopausal breast cancer patient includes inducing a syngeneic, orthotopic, ER+ mammary tumor with subsequent surgical removal by radical mastectomy, then a repeated chemotherapy regimen, followed by long-term aromatase inhibitor treatment in an ovariectomized (modeling post-menopause) female mouse.

## Materials And Methods

### Animals

Nulliparous, female, 8- to 9-week old Balb/c mice (Charles River, Wilmington, MA, USA) were housed 5/cage and acclimated to the temperature-controlled (22 ± 1°C) vivarium under a 14:10 light:dark cycle (lights off at 14:00 h). Rodent chow (Harlan 7912) and water were available *ad libitum* throughout the study. Cotton nestlets and plastic huts were provided for nesting and enrichment, and mice were acclimated to handling twice/week. All experiments were approved by the Ohio State University Institutional Animal Care and Use Committee and carried out in accordance with the National Institutes of Health Guide for the Care and Use of Laboratory Animals (NRC, 2011). All efforts were made to minimize animal suffering and to reduce the number of mice used.

### Experimental Design

**Figure 1:**
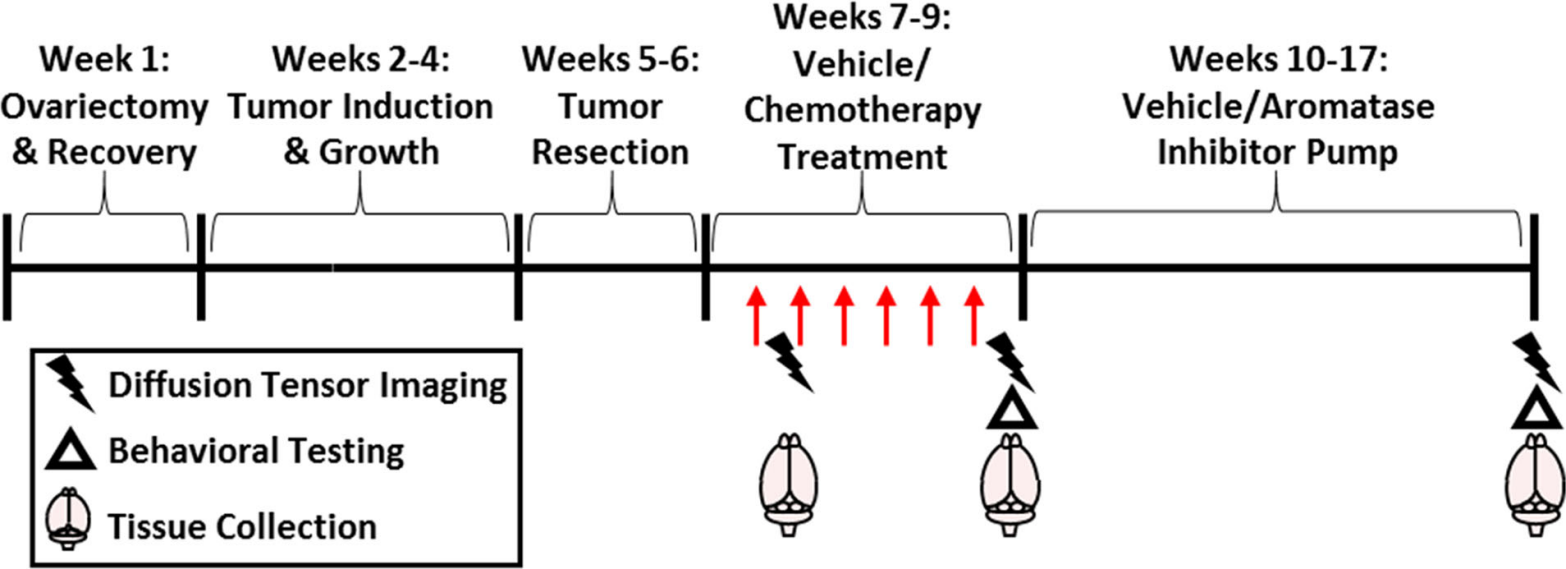
Experimental Overview. Mice were ovariectomized followed by 1 week of recovery and then surgical mammary tumor induction. After 1.5 cm diameter tumor growth, the tumor was resected. Paclitaxel chemotherapy or vehicle (30 mg/kg; i.p.; 5-7 doses) with/without subsequent letrazole (10 µg/day over 57 days) or control treatment was administered. Brain imaging and tissue collection occurred after the first dose of chemotherapy, after the final dose, or after letrozole treatment. Behavioral testing was recorded after chemotherapy or letrozole treatments.

All mice were ovariectomized (OVX) under isoflurane vapors, and following 1 week of recovery, mammary tumors were induced. Tumors were allowed to grow (approximately 3 weeks) and then were surgically resected. Mice were then separated into one of four groups: (1) vehicle + control, (2) vehicle + aromatase inhibitor, (3) chemotherapy + control, (4) chemotherapy + aromatase inhibitor. After 1 dose of chemotherapy, 1 cohort of treatment-balanced mice underwent diffusion weighted imaging (DWI). After 6 rounds of chemotherapy, behavioral tests, DWI, and gene expression analyses were conducted in a second cohort. A third cohort had 40 days of aromatase inhibitor treatment, then cognitive behavioral tests and DWI were conducted (**Figure 1**).

### Cells

The murine, mammary, non-metastatic 67NR cancer cell line was used in this study. Importantly, this cell line is ER+ consistent with the majority (~80%) of breast tumors in women (40). The cells were grown in DMEM with 10% FBS, 2 mM _L_-glutamine, 1 mM non-essential amino acids, and 5mL Penn-Strep antibiotic at 37°C with 5% CO_2_ as previously described (41–43). Cells were harvested and suspended 1:1 in matrigel (47743-706, VWR, Radnor, PA, USA) immediately prior to use.

### Tumor Survival Mouse Model

Our tumor “survivor” model of breast cancer was induced 1 week after surgical ovariectomy in all mice using methods previously reported (43, 44). Briefly, tumors were surgically induced in all mice under isoflurane vapor anesthetization by injecting 1 x 10^6^ 67NR mammary tumor cells in matrigel, described above, into the 4^th^ mammary fat pad. This procedure results in an *in situ* primary mammary carcinoma (45) that does not metastasize (46), which eliminates the need for immunocompromised mice. Body mass and tumor dimensions were measured twice/week. Mice that failed to develop a tumor were removed from the study. When the tumors reached 15 x 10 mm in size (approximately 3 weeks), a modified radical mastectomy procedure was used to completely remove the tumor. Mice were anesthetized and tumors were surgically removed along with mammary tissue, fat, and inguinal lymph nodes where necessary. Tumors weighed 0.96 ± 0.32 at resection, on average, and there were no statistically significant differences between groups (p > 0.05). Buprenorphine (0.05 mg/kg; s.c.) was administered immediately after surgery, and every 6-12 h over 3 days as needed. Complete tumor resection was verified at necropsy and mice with recurrent tumors were excluded from analyses. A pilot study (n=5-6/group) was conducted to confirm the menopausal-like state caused by OVX. OVX significantly reduced circulating estrogen levels (p > 0.05) and halted estrous cycling (p > 0.01) approximately 3 weeks after ovariectomy (**Supplementary Figure 1**).

### Drug Treatments

The common breast cancer chemotherapeutic drug, paclitaxel (T7191, Sigma-Aldrich, St. Louis, MO, USA), was administered in a series of six intraperitoneal injections (30 mg/kg body mass) or vehicle every other day as previously described (47–50) unless otherwise noted. The regimen was modeled after the 4-8 doses of paclitaxel separated by 1-3 weeks for breast cancer patients. Every other day dosing for this regimen was determined using mouse lifespan calculations (10 human years ~ 2 mouse months) (48). One week after the last chemotherapy injection, mini-osmotic pumps (7223, model 2006; Alzet, Cupertino, CA, USA) containing either the aromatase inhibiting (reduces estrogen) drug, letrozole (Sigma-Aldrich), or control were surgically implanted subcutaneously. After 40 days of treatment, Alzet pumps were replaced with fresh letrozole containing pumps. Letrozole (L6545, Sigma-Aldrich, St. Louis, MO, USA) was dissolved in 10% dimethyl sulfoxide (DMSO) in PBS. Each mouse received 10 µg letrozole/day (51) over 57 days. Aromatase inhibitors are used frequently in the treatment of breast cancer and letrozole is the most studied aromatase inhibitor in mice (52). The dosage was chosen based on its effectiveness in reducing mammary tumor growth in mice, the goal of aromatase inhibitor therapy in humans, and previous studies (51, 53, 54). The duration of treatment is a scaled down version of clinical treatment based on mouse lifespan calculations (10 human years ~ 2 mouse months).

### Diffusion Weighted Imaging

One day after 1 round of chemotherapy, 1-2 days after 6 rounds of chemotherapy, or after 57 days of letrozole treatment (see **Figure 1**), mouse brains were imaged *in vivo*. Diffusion weighted imaging (DWI) was conducted at The Ohio State University small animal imaging core (Columbus, OH, USA) using a 9.7 T BioSpec 94/30 horizontal bore magnet (Bruker, Billerica, MA, USA) with a mouse brain phased array coil and ParaVision™ 5.1 software. Mice were anesthetized with isoflurane. Images were acquired with a spin-echo echo-planar-imaging (EPI) pulse sequence with the following acquisition protocol: TR/TE = 400/17.8 ms, 8 EPI segments, and 20 non-collinear gradient directions with a single *b*-value shell at 900 s/mm^2^, and one image with a *b*-value of 0 s/mm^2^ (b0). Geometrical parameters were six slices, each 0.313 mm thick (brain volume) with an in-lane resolution of 0.15x0.15 mm^2^ (matrix size 112 x 100; FOV 30 mm^2^). Each acquisition took approximately 44 min, and the entire MRI protocol lasted about 1 h 13 min. The body temperature and respiration rates of mice were monitored using the Monitoring and Gating SAII system (Small Animal Instruments, Inc. Stony Brook, NY, USA) throughout imaging.

DWI images were analyzed to produce maps of fractional anisotropy (FA), apparent diffusion coefficient (ADC), linear diffusivity (L1), and radial anisotropy (RA) with procedures previously described (55, 56) using MATLAB and MedINRIA (1.9.0^1^) software. Each image was screened for movement artifacts, and acquisition points with motion artifacts were eliminated from further analysis. All images were aligned and registered to a 3D Mouse Brain Atlas^©^ with 134 segmented and annotated brain regions (Ekam Solutions; Boston, MA) for voxel- and region based statistical comparisons (55) using MIVA software (http://ccni.wpi.edu). For each mouse, the b0 image was registered with the b0 template using a six-parameter rigid-body transformation. The co-registration parameters were then applied to the DWI-indexed maps for the different indices of anisotropy. Normalization was performed on the maps because they provided the most detailed visualization of brain structures, and these normalizations were applied to all DWI indexed maps and smoothed with a 0.3-mm Gaussian kernel. The “nearest neighbor” option was used following registration and normalization to ensure FA and RD values were not significantly affected by the pre-processing steps.

All image transformations and statistical analyses were carried out using EVA (Ekam Visualization and analysis, Ekam Solutions LLC, Boston, MA) and in-house MATLAB^®^ based software. For each mouse, the B0 image was co-registered with the MRI brain atlas using a 9 degree affine transformation [T]. A completely segmented map-file for each subject was generated using [T^-1^] matrix. While generating map file nearest-neighbor interpolation was used to avoid mixing of segmented ROI’s. The statistical parameters (mean, median, std dev etc.) for each ROIs and for each indices were computed based on this map file and information was exported to comma separated value (CSV) file. For each subject, each ROI and each diffusion indices mean, std deviation, mode, minimum and maximum values were reported. Statistical differences in measures of DWI between experimental groups were determined using a nonparametric Mann-Whitney U Test (alpha set at 5%). The formula below was used to account for false discovery from multiple comparisons.


$$P_{\left(i\right)}\leq \frac{i}{V}\;\frac{q}{c(V)}$$

P(i) is the p value based on the t test analysis. Each of 134 ROIs (i) within the brain containing (V) ROIs was ranked in order of its probability value. The false-positive filter value q was set to 0.2 and the predetermined c(V) set at unity.

### Behavioral Testing

Total locomotion in a novel environment was assessed using the open field test. Mice were placed into the corner of a 40.6 x 40.6 cm photobeam arena (San Diego Instruments, San Diego, CA, USA) that was lightly covered with corncob bedding. Mice were allowed to freely explore for 15 min. The apparatus was cleaned with 70% ethanol between each mouse. Locomotor measures were analyzed using PAS Data Reporter (San Diego Instruments) and reported as beam breaks.

Working memory and speed in a novel environment were tested during the spontaneous alternation test. Each mouse was placed into the center of a Y-maze consisting of 3 equal-length gray acrylic arms (40 L x 8 W x 15 H cm) at angles of 120° and allowed to explore the entire maze for 3 min. Each test was recorded using an overhead camera and tracked using ANY-Maze video tracking software (Stoelting Co., Sand Diego, CA USA). A successful alternation was defined as successive entries into each of the 3 arms in any order. The percent spontaneous alternation was calculated as the number of successful alternations divided by the total number of possible alternations and multiplied by 100. Locomotor speed (m/s) was tracked using the ANY-Maze software.

### Tissue Collection

Tissues were collected two days after one dose of chemotherapy, one day after the final dose of chemotherapy, or after 7 weeks of letrozole treatment. Mice were rapidly decapitated, blood was collected using heparinized tubes, and specific brain regions (hippocampus and frontal cortex) were immediately dissected out and frozen on dry ice. Spleens and tumors were also collected and weighed.

### Plasma Cytokine Concentrations

As DWI changes were only significant directly after the final dose of chemotherapy, we focused inflammation analyses on this timepoint rather than after 1 dose of chemotherapy or after aromatase inhibitor treatment. At this timepoint, plasma cytokines were measured using a custom 7-plex Meso-Scale Discovery (MSD) immunoassay plate (U-PLEX Biomarker Group 1 (ms) assay, SECTOR, MSD Cat. No. K15069L-2) according to the manufacturer’s instructions. This assay measured protein levels of interferon gamma (*IFNγ*), interleukin 1 beta (*IL-1β*), interleukin 2 (*IL-2*), interleukin 6 (*IL-6*), interleukin 10 (*IL-10*), chemokine (C-X-C motif) ligand 1 (*CXCL1*), and tumor necrosis factor alpha (*TNFα*). Intraplate variability for all analytes was <5%.

### Gene Array

Total RNA was extracted from the brain hippocampus and frontal cortex of vehicle- or paclitaxel-treated mice using Qiagen RNeasy Mini Kits (CA, USA). RNA concentrations and quality were determined (NanoDrop, DE, USA), then RNA from both regions were combined equally. Five hundred ng of isolated RNA was reverse transcribed using the RT^2^ First Strand Kit (Qiagen, Cat. No. 330231, Frederick, MD, USA). Expression of eighty-four genes associated with mouse innate and adaptive immune responses was analyzed simultaneously using the RT^2^ Profiler PCR array (Qiagen, Cat. No. PAMM-032ZE). RT^2^ SYBR Green qPCR master mix (Qiagen, Cat. No. 330522) was used following the manufacturer’s instructions. Gene expression was normalized using the geometric mean of a panel of housekeeping genes including Beta actin (*Actb*), Beta-2 microglobulin (*B2m*), glyceraldehyde 3-phosphate dehydrogenase (*Gapdh*), Beta-glucoronidase (*Gusb*), and Heat shock protein HSP 90-beta (*Hsp90ab1*). Relative gene expression of individual samples was calculated by the comparative C_T_ method (2^-ΔΔCT^) and results are shown as fold change from the average vehicle expression value. As the sample size in this gene array was low, we conducted validation RT-qPCR of *Icam* in the hippocampus and frontal cortex, separately. We found a significant increase in *Icam* expression in the frontal cortex (p < 0.05) but not the hippocampus (p > 0.1) (**Supplementary Figure 2**), suggesting that the frontal cortex was driving the increase with chemotherapy in the gene array.

### Immunohistochemistry

One to 2 days following the final dose of paclitaxel chemotherapy, mice were anesthetized and perfused using 4% paraformaldehyde. Briefly, brains were placed in 4% paraformaldehyde overnight and then into a 30% sucrose solution for 3-4 days. Brains were frozen, cut at 10µm on a cryostat, and mounted. Sections were immunolabeled for Iba1 or GFAP as previously described (Invitrogen) (57). Next, three images from hypothalamus (paraventricular nucleus and lateral hypothalamic nucleus) and hippocampus (CA3 region) were collected for each brain, and the immunoreactive area of GFAP and Iba1 of each section was quantified using image analysis (Image J). Area data was divided by scan area and the data from the 3 sections for each brain were averaged and group means compared (**Supplementary Figure 3**). Further immunohistochemistry was conducted to investigate white matter specifically. We stained for myelin (eriochrome cyanine) and oligodendrocytes (glutathione s-transferase pi – GSTpi). We could not interpret the results due to the neurostructural abnormalities (e.g., lack of corpus callosum) endemic to the Balb/c mouse strain (58–60). These developmental abnormalities were observed in mice regardless of treatment group. Thus, we could not proceed with statistical analyses or subsequent conclusions based on these immunohistochemical data.

### Statistical Analyses

Statistical analyses of behavioral, gene expression, imaging, and cytokine data were performed using unpaired, parametric, two-tailed t-tests (post-chemotherapy) or one-way ANOVA (post-AI) followed by Tukey’s correction HSD or multiple Student’s *t*-tests controlling for multiple comparisons based on a *priori* hypotheses (Statview version 5.0.1 software, Scientific Computing, Cary, NC, USA). Nonparametric Mann-Whitney U tests were used when the assumptions of normality and equal variances were not met. Data were considered statistically significant when p ≤ 0.05 and are presented as mean ± standard error of the mean (SEM).

## Results

### Chemotherapy Reduced Locomotor Activity

**Figure 2:**
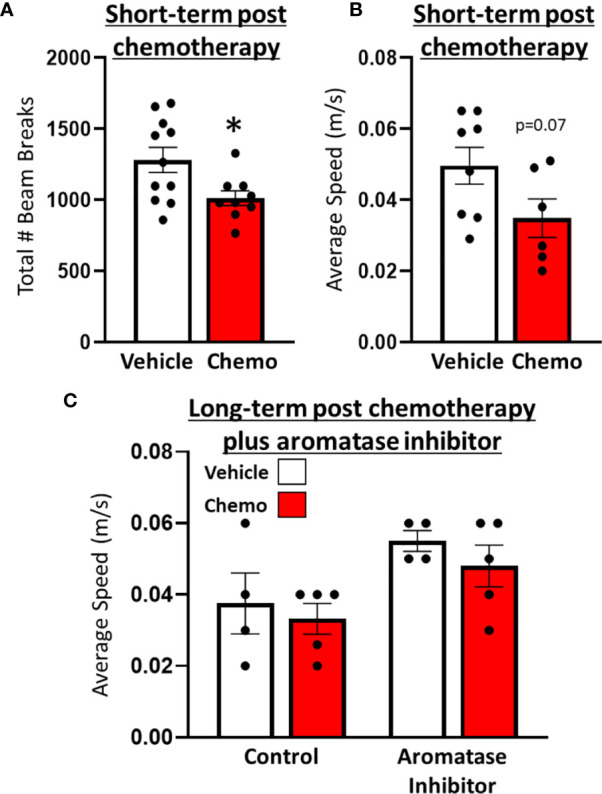
Paclitaxel chemotherapy induced a transient decrease in locomotor activity following the final dose in survivor mice. **(A)** Locomotion in an open field test 1 day following the final dose of paclitaxel (n=9) or vehicle (n=11). **(B)** Average speed (m/s) in a spontaneous alternations test following the final dose of paclitaxel (n=6) or vehicle (n=8). **(C)** Average speed (m/s) in a spontaneous alternations test following aromatase inhibitor treatment (n=4/group). Unpaired parametric two-tailed t tests were used for the short-term time point, and a one-way ANOVA was used for the long-term time point. Nonparametric Mann-Whitney U tests were used when the assumptions of normality and equal variances were not met. *p<0.05.

One day after the final dose of paclitaxel, mice treated with paclitaxel had reduced locomotor activity compared with vehicle-treated mice in the open field test (**Figure 2A**, t_16_ = 2.62, p = 0.02). Similarly, speed in the spontaneous alternations test approached significantly different between mice treated with 6 doses of paclitaxel or vehicle (**Figure 2B**, *t_11_* = 1.98, *p* = 0.07). Following subsequent chronic letrozole treatment, speed recovered (**Figure 2C**, *F_1,18_* = 0.04, *p* = 0.85), but letrozole moderately increased speed in the spontaneous alternations test (**Figure 2C**, *F_1,18_* = 4.00, *p* = 0.06). No differences in percent spontaneous alternations (working spatial memory) were observed at either time point (p > 0.05; **Supplementary Figure 4**).

### Chemotherapy Induced Widespread Changes in DWI Anisotropy

**Figure 3:**
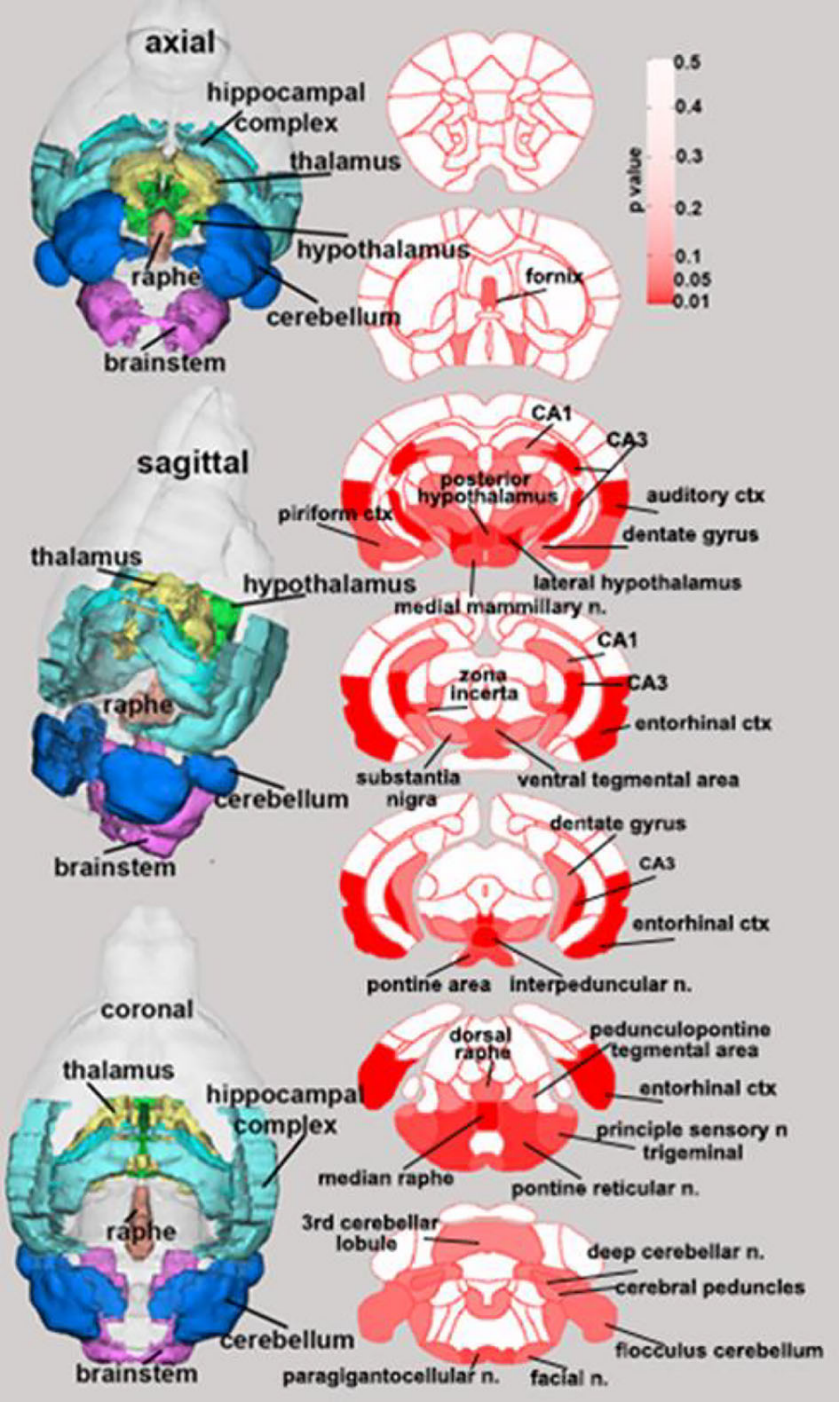
Paclitaxel chemotherapy consistently increased fractional anisotropy following the final dose in survivor mice. Axial, sagittal, and coronal views of fractional anisotropy probability maps calculated from diffusion weighted imaging are shown. Paclitaxel n=8, vehicle n=8. Unpaired parametric two-tailed t tests were used for statistical analyses. Nonparametric Mann-Whitney U tests were used when the assumptions of normality and equal variances were not met.

Few changes in fractional anisotropy (FA), apparent diffusion coefficient (ADC), radial anisotropy (RA), or linear diffusivity (L1) occurred 1 day after 1 dose of chemotherapy (**Supplementary Tables 1**–**4**) or following repeated chemotherapy plus chronic aromatase inhibitor treatment (**Supplementary Tables 9**–**12**). In contrast, 1 day after the final dose of the paclitaxel regimen, chemotherapy increased FA and decreased ADC throughout many areas of the brain, including the hippocampus, midbrain, medulla, pons, hypothalamus, thalamus, amygdala, and cerebellum (**Figure 3** and **Supplementary Tables 5**–**8**). FA data are represented by probability heat maps that illustrate the statistical differences between mice treated with 6 doses of paclitaxel compared to mice treated with vehicle (**Figure 3**). Mice exposed to 6 doses of chemotherapy had increased FA in numerous brain regions that regulate various behaviors, including the CA3 region of the hippocampus and throughout the hypothalamus (summarized in **Table 1**, full analyses in **Supplementary Table 5**). Conversely, ADC was largely decreased throughout the brain in mice treated with chemotherapy (summarized in **Table 2**, full analyses in **Supplementary Table 6**).

**Table 1:** Brain regions where paclitaxel significantly increased fractional anisotropy.

Brain Area	p-value	Brain Area	p-value	Brain Area	p-value	Brain Area	p-value
interpeduncular area	0.001	medial mammillary area	0.01	basal amygdaloid area	0.02	cerebellar nuclear area	0.04
median raphe area	0.001	pyramidal tracts	0.02	zona incerta	0.02	flocculus cerebellum	0.04
CA3	0.002	principal sensory nucleus trigeminal	0.02	reticular thalamic area	0.02	anterior hypothalamic area	0.04
lateral caudal hypothalamic area	0.004	lateral rostral hypothalamic area	0.02	ventral medial hypothalamic area	0.03	medial amygdaloid area	0.05
entorhinal cortex	0.004	ventral tegmental area	0.02	fornix	0.03	dentate gyrus	0.05
posterior hypothalamic area	0.005	olivary complex	0.02	dorsal raphe	0.03	anterior pretectal thalamic area	0.05
pontine reticular nucleus oral	0.007	medial lemniscus	0.02	crus of ansiform lobule	0.03	cuneate area	0.05
lateral lemniscus	0.01	ambiguus area	0.02	paramedian lobule	0.03	cerebral peduncle	0.05
anterior thalamic area	0.01	lateral paragigantocellular area	0.02	decussation superior cerebellar peduncle	0.04	spinal trigeminal nuclear area	0.05
pontine area	0.01	caudal piriform cortex	0.02	facial nucleus	0.04		

(For all regions Vehicle < Paclitaxel). Unpaired parametric two-tailed t tests were used for statistical analyses. Nonparametric Mann-Whitney U tests were used when the assumptions of normality and equal variances were not met.

**Table 2:** Brain regions where paclitaxel significantly decreased apparent diffusion coefficient.

Brain Area	p-value	Brain Area	p-value	Brain Area	p-value	Brain Area	p-value
posterior hypothalamic area	0.002	pontine reticular nucleus oral	0.02	pedunculopontine tegmental area	0.02	entorhinal cortex	0.04
central amygdaloid area	0.002	pontine reticular nucleus caudal	0.02	mesencephalic reticular formation	0.03	infralimbic cortex	0.04
medial lemniscus	0.003	ventral tegmental area	0.02	parabrachial area	0.03	globus pallidus	0.04
median raphe area	0.009	extended amygdala	0.02	lateral septal area	0.03	medial septal area	0.04
medial mammillary area	0.01	reticulotegmental nucleus	0.02	crus of ansiform lobule	0.03	lateral amygdaloid area	0.05
anterior pretectal thalamic area	0.01	lateral posterior thalamic area	0.02	insular caudal ctx	0.03	parafascicular thalamic area	0.05
dorsal raphe	0.02	periaqueductal gray	0.02	dentate gyrus	0.03	caudate putamen	0.05
fornix	0.02	dorsal medial hypothalamic area	0.02	lemniscal area	0.03		

(For all regions Vehicle > Paclitaxel). Unpaired parametric two-tailed t tests were used for statistical analyses. Nonparametric Mann-Whitney U tests were used when the assumptions of normality and equal variances were not met.

### Chemotherapy Induced Peripheral Inflammation

**Figure 4:**
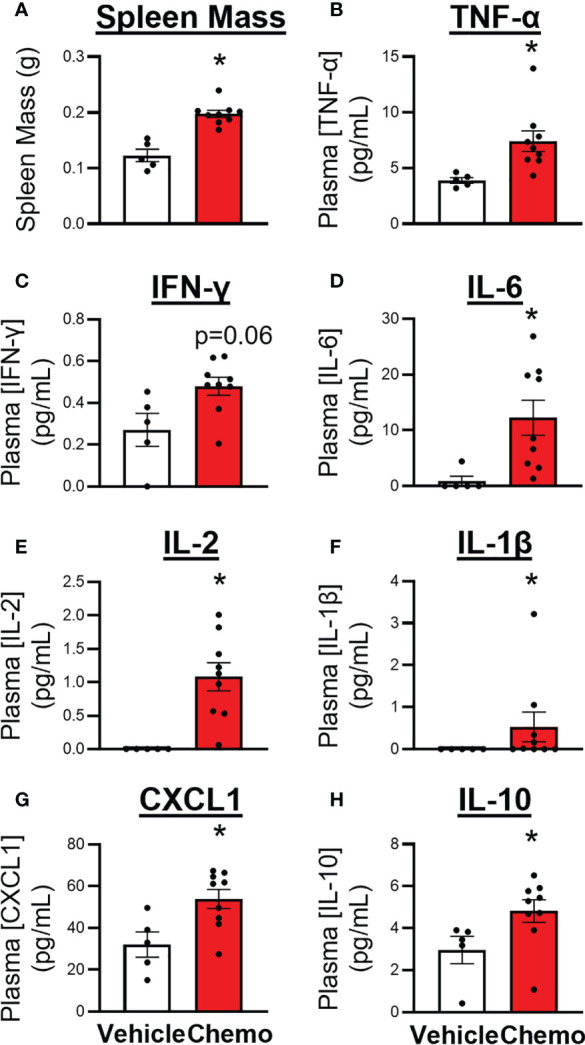
Paclitaxel chemotherapy increased circulating inflammatory markers following the final dose in survivor mice. **(A)** Spleen mass, plasma levels of pro- and anti- inflammatory cytokines, **(B)** TNFα, **(C)** IFNγ, **(D)** IL-6, **(E)** IL-2, **(F)** IL–1β, **(G)** CXCL1, and **(H)** IL-10. Paclitaxel n=9, vehicle n=5. Unpaired parametric two-tailed t tests were used for statistical analyses. Nonparametric Mann-Whitney U tests were used when the assumptions of normality and equal variances were not met. *p<0.05.

Spleens and plasma were collected from mice 1 day after the 6^th^ injection of chemotherapy. Chemotherapy treatment induced splenomegaly (**Figure 4A**, *t_7_* = 5.81, *p* = 0.0007), increased circulating inflammatory proteins including TNFα (**Figure 4B**, χ^2^ = 8.22, *p* = 0.004), IFNγ (**Figure 4C**, *t*_6_ = 2.34, *p* = 0.06), IL-6 (**Figure 4D**, χ^2^ = 6.91, *p* = 0.009), IL-2 (**Figure 4E**, χ^2^ = 9.41, *p* = 0.002), IL-1β (**Figure 4F**, χ^2^ = 3.77, *p* = 0.05), CXCL1 (**Figure 4G**, *t_9_* = 2.89, *p* = 0.02), and increased the anti-inflammatory protein IL-10 (**Figure 4H**, χ^2^ = 5.44, p = 0.02).

### Chemotherapy Altered Hippocampal and Frontal Cortex Inflammatory Gene Expression

**Figure 5:**
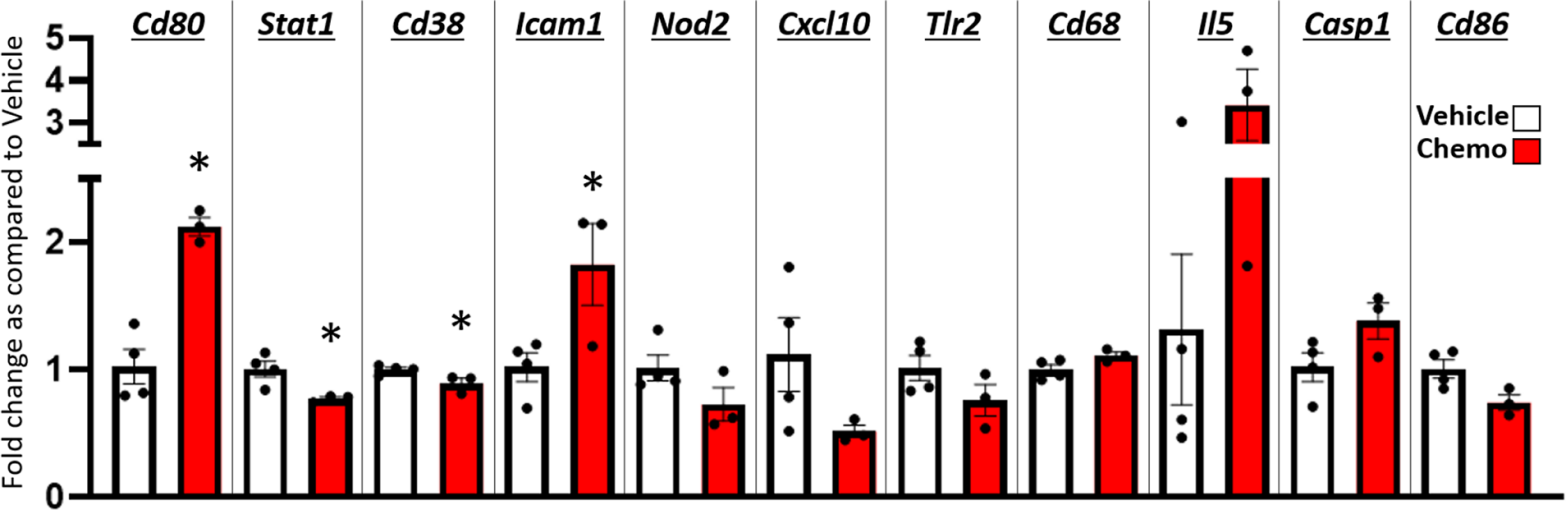
Paclitaxel chemotherapy dynamically alters gene expression in the hippocampus and hypothalamus combined. Relative gene expression shown as fold change as compared to vehicle average of multiple inflammation related genes. Paclitaxel n=3, vehicle n=4. Unpaired parametric two-tailed t tests were used for statistical analyses. Nonparametric Mann-Whitney U tests were used when the assumptions of normality and equal variances were not met. *p<0.05.

Based on the widespread changes in anisotropy suggesting neuroinflammation (55) in regions that regulate locomotion following 6 doses of chemotherapy, a quantitative PCR array for innate and adaptive immune response genes was conducted in combined hippocampus/frontal cortex tissues (**Figure 5**). Four genes were significantly changed after 6 doses of chemotherapy (p < 0.05) such that *Cd80* and *Icam1* expression was significantly increased with chemotherapy whereas *Stat1* and *Cd38* expression was significantly decreased with chemotherapy. An additional seven genes approached significant changes with chemotherapy treatment (p < 0.1) such that *Cd68*, *Il5*, and *Casp1* expression was increased with chemotherapy and *Nod2, Cxcl10, TLR2*, and *Cd86* expression was decreased. Further, chemotherapy did not alter percent Iba-1 and GFAP area, measured *via* immunohistochemistry, in the hippocampus and hypothalamus (p > 0.1; **Supplementary Figure 3**).

## Discussion

As breast cancer patients have a combination of clinical factors affecting their health, treatment, and recovery, comprehensive rodent models with greater translational value are needed to accurately identify biological targets for intervention. The present mouse model, to our knowledge, is the first to depict a typical breast cancer patient using an ovariectomy (post-menopausal woman), an orthotopic, syngeneic, ER+ mammary tumor, tumor resection, chemotherapy, and then aromatase inhibitor treatment. This inclusive model is particularly useful for studying synergistic or additive effects of these independent factors. Indeed, breast cancer patients do not face singular biological insults, they face multiple complex factors, often simultaneously. Additional factors typical of the cancer experience, including stressors, advanced age, and infections, could also be added to this model depending on the specific scientific questions being investigated. This model was created to be dynamic and expandable with the capability to investigate a multitude of hypotheses in various fields of study (e.g., aging, radiation, stressors, surgical complications).

In this study, we assessed the extent to which a cumulative breast cancer model recapitulates the fatigue observed in many breast cancer patients. Fatigue in humans has multiple components, including reduced locomotor activity, motivation, and cognition. Chemotherapy did not significantly affect cognition-based behavior in a spontaneous alternations test (percent spontaneous alternations – **Supplementary Figure 1**) but did significantly reduce locomotor activity after the final dose of chemotherapy (**Figure 1**). Locomotor activity assessment in cancer patients often uses wrist actigraphy and smartwatches to track movement, which is similar to our measurement of movement in the open field test (61–63). The timing of the observed locomotor activity reduction, which occurred shortly after chemotherapy, was consistent with other human and rodent studies (7, 49, 64, 65). Specifically, paclitaxel (used in this model), induces fatigue in humans and reduces locomotor activity in rodents (49, 65, 66). Of note, our previous work using ovary-intact, tumor-free mice indicates that paclitaxel induces central but not muscle-related reduced locomotor activity (49). We have also previously demonstrated that tumor resection reduces locomotor activity on its own (43), indicating that in the present study chemotherapy may exacerbate tumor resection-induced reduced locomotor activity. Additional behavioral testing is warranted to dissect the potential cognitive, memory, and motivational components of fatigue in the present comprehensive model.

As behavioral comorbidities, including fatigue, have been previously associated with white matter structural abnormalities in women (18), we used diffusion weighted imaging to evaluate brain structure changes after various aspects of the treatment regimen. To our knowledge, this is the first study of MRI in mice treated with chemotherapy. Minimal changes after 1 dose of paclitaxel were observed. Whereas, after the final dose of paclitaxel, imaging analysis indicated a transient global chemotherapy-induced increase in fractional anisotropy (FA) and decrease in apparent diffusion coefficient (ADC) in the brain. When mice were allowed to recover from chemotherapy and receive an aromatase inhibitor, these FA and ADC alterations resolved. Specifically, FA and ADC alterations were absent after 1 dose, although previous work indicates that reduced locomotor activity is already detectable at this time (49), suggesting that FA and ADC may not directly relate to reduced locomotor activity. While DWI was used to specifically assess white matter changes within the brain, broad and diffuse FA and ADC changes were observed after the 6^th^ dose of paclitaxel, likely indicating widespread inflammation throughout white and gray matter of the brain. Indeed, these measures of anisotropy are reported to reflect alterations in gray matter microarchitecture associated with neuroinflammation following brain injury (55). In support of this interpretation, we have previously observed transient neuroinflammation in otherwise naïve mice treated with chemotherapy (47, 48). Immunohistochemistry was conducted to further investigate specific white matter changes. As Balb/c mice have neurostructural abnormalities (e.g., lack of corpus callosum) (58–60) the results of myelin and oligodendrocyte staining were not interpretable. These developmental abnormalities were observed in mice regardless of treatment group. Thus, we could not proceed with statistical analyses or subsequent conclusions based on these immunohistochemical data. Many brain regions affected by chemotherapy are part of the ascending reticular activating system which is involved in consciousness. Future studies will focus on resting-state functional connectivity analysis of functional MRI (fMRI) to better understand how chemotherapy globally affects communication between brain areas.

Inflammatory pathways are involved in a host of behavioral and cognitive disorders, including fatigue and depression in humans and rodents (67). Consistent with these studies, the current study has recapitulated the peripheral inflammation coincident with reduced locomotor activity as well as demonstrated that chemotherapy also drives widespread changes in brain anisotropy in this more comprehensive breast cancer model. In addition, our pooled hippocampal and frontal cortex samples showed a modest number of genes that contribute to the migration, function, and/or recognition of antigen by immune cells were altered directly after chemotherapy treatment. Inflammatory differences between vehicle- and chemotherapy-treated mice using this comprehensive model may be less dramatic than in simpler models that only administer chemotherapy as the vehicle controls in this study received multiple inflammatory insults (OVX surgery, tumor induction, tumor resection) (68, 69). The immunohistochemical analysis of Iba-1 and GFAP labeling, markers of microglia and astrocytes, respectively, remained comparable between groups 1-2 days after the final dose of chemotherapy. We have previously shown transient neuroinflammation with this chemotherapy regimen in ovary-intact, tumor free mice (47–49, 64). This neuroinflammation is present at 6 hours after the final dose but not 72 hours. Given the cross-sectional nature of this study and the dynamic activation of glial cells, it is possible that we missed the glial morphological activation state as measured by Iba-1 and GFAP staining. This investigation of neuroinflammation is not comprehensive and future studies will investigate neuroinflammation overtime by measurement of a more comprehensive spread of inflammatory markers, including CD68 and IL-1 protein.

Coincident with inflammation and reduced locomotor activity, the present DWI data indicate some areas of high structural alterations, including the hippocampal CA3 region, the auditory and entorhinal cortices of the temporal lobe, the interpeduncular nucleus of the midbrain tegmentum, the median raphe and pontine reticular nucleus of the pons, and the posterior hypothalamus. In addition to locomotor activity, these brain regions regulate other behaviors that can be impaired in cancer patients during and after treatment (e.g., cognition, mood).

This study has some limitations. First, the transcriptional and immunohistochemistry analyses sample sizes were relatively low. Larger sample sizes were used for behavior, DWI, and circulating inflammatory marker analyses. Further studies are needed to delineate the underlying mechanisms of global anisotropy changes seen with chemotherapy in the DWI data. Also, neuroinflammation was examined cross-sectionally and at a transcriptional, not protein, level. Further tests are needed to expand upon the potential cognitive, motivational, and locomotive behavioral consequences in this model. Notably, breast cancer patients often receive a combination of chemotherapeutics as well as radiation, which is not accounted for in this study and warrants future investigations. Future studies could expand this model to incorporate multiple chemotherapeutics and radiation. Finally, this study uses young (~4.5 – 6.5 months at the time of behavioral and biological analyses) ovariectomized mice, whereas a natural menopause would add even greater clinical translatability (70, 71).

Taken together, this study establishes a useful and comprehensive rodent model of breast cancer that combines menopausal status, tumor growth, surgery, chemotherapy, and aromatase inhibitors in sequence and results in inflammation, neuroimaging alterations, and reduced locomotor activity consistent with many breast cancer patients. This model will continue to be advantageous for investigating how multiple complex biological aspects of the breast cancer experience interact to cause the cognitive and behavioral comorbidities that reduce quality of life for breast cancer patients and their loved ones. Furthermore, the neurobiological mechanisms and associated brain imaging that are established using this rodent model could be used to infer the appropriate intervention targets based on comparable imaging in patients.

## Data Availability Statement

The original contributions presented in the study are included in the article/**Supplementary Material**. Further inquiries can be directed to the corresponding author.

## Ethics Statement

All experiments were approved by the Ohio State University Institutional Animal Care and Use Committee and carried out in accordance with the National Institutes of Health Guide for the Care and Use of Laboratory Animals (NRC, 2011). All efforts were made to minimize animal suffering and to reduce the number of mice used.

## Author Contributions

LP designed experiments. KR, LP, and LO analyzed and interpreted data. LO, KR, and LP wrote manuscript. PK and CF analyzed and interpreted diffusion weighted imaging data. DM provided data for immunohistochemistry. All authors reviewed the final manuscript. All authors contributed to the article and approved the submitted version.

## Funding

This work was supported by The Ohio State University Medical Center, a CCTS core grant UL1TR001070, and NIH R01CA21690 (LP).

## Conflict of Interest

The authors declare that the research was conducted in the absence of any commercial or financial relationships that could be construed as a potential conflict of interest.

## Publisher’s Note

All claims expressed in this article are solely those of the authors and do not necessarily represent those of their affiliated organizations, or those of the publisher, the editors and the reviewers. Any product that may be evaluated in this article, or claim that may be made by its manufacturer, is not guaranteed or endorsed by the publisher.

## Supplementary Material

Supplementary Figure 1 Ovariectomy induces menopausal-like state in Balb/c mice. **(A)** Ovariectomy halts estrous cycling. **(B)** Ovariectomy significantly decreases circulating estrogen concentrations.

Supplementary Figure 2 Paclitaxel chemotherapy increased *Icam* expression in the frontal cortex but not the hippocampus following the final dose in survivor mice. **(A)** Hippocampus, **(B)** Frontal cortex. Unpaired parametric two-tailed t tests were used for statistical analyses. Nonparametric Mann-Whitney U tests were used when the assumptions of normality and equal variances were not met. *p<0.05

Supplementary Figure 3 Paclitaxel chemotherapy does not alter Iba-1 or GFAP percent area in the hippocampus or hypothalamus following the final dose in survivor mice. **(A)** Hippocampal Iba-1% area, **(B)** Hypothalamic Iba-1% area, **(C)** Hippocampal GFAP % area, **(D)** Hypothalamic GFAP % area. Paclitaxel n=4, vehicle n=3. Unpaired parametric two-tailed t tests were used for statistical analyses. Nonparametric Mann-Whitney U tests were used when the assumptions of normality and equal variances were not met. *p<0.05.

Supplementary Figure 4 Neither paclitaxel chemotherapy nor aromatase inhibitor treatment affected percent spontaneous alternations. **(A)** Percent spontaneous alternations in the spontaneous alternations test following the final dose of paclitaxel (n=8/group). **(B)** Percent spontaneous alternations in the spontaneous alternations test following aromatase inhibitor treatment. Aromatase inhibitor n=5, control n=4. Unpaired parametric two-tailed t tests were used for statistical analyses. Nonparametric Mann-Whitney U tests were used when the assumptions of normality and equal variances were not met. *p<0.05.
