# Supplementary figures and images for: Paclitaxel Chemotherapy Elicits Widespread Brain Anisotropy Changes in a Comprehensive Mouse Model of Breast Cancer Survivorship: Evidence From In Vivo Diffusion Weighted Imaging
Source: Front Oncol. 2022 Mar 23;12:798704. doi: 10.3389/fonc.2022.798704 (PMC8984118; doi:10.3389/fonc.2022.798704)

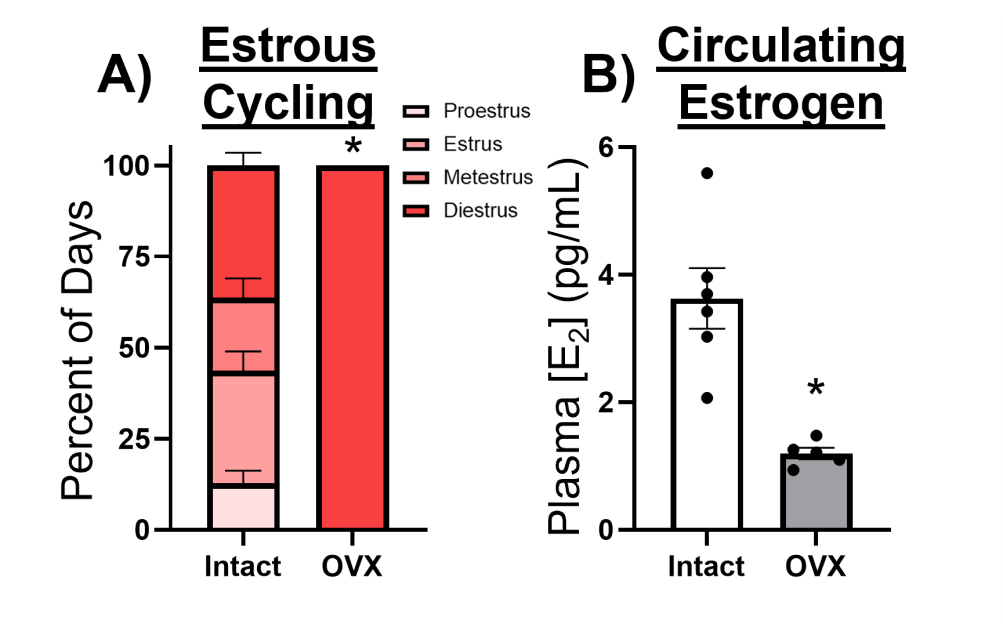

Supplement: Supplementary Figure 1 — Ovariectomy induces menopausal-like state in Balb/c mice. (A) Ovariectomy halts estrous cycling. (B) Ovariectomy significantly decreases circulating estrogen concentrations. [file Image_1.tiff]

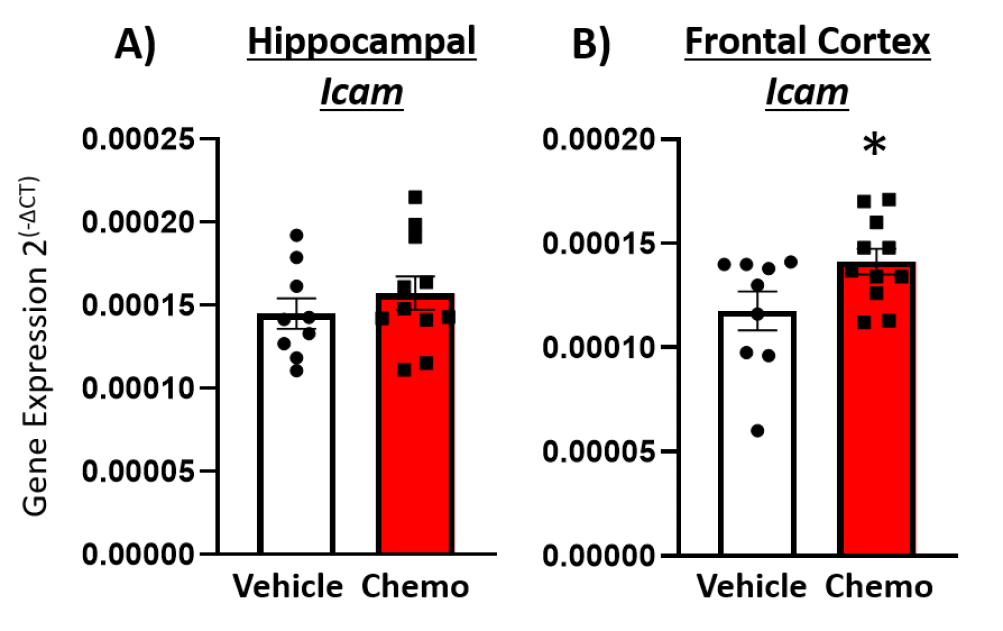

Supplement: Supplementary Figure 2 — Paclitaxel chemotherapy increased Icam expression in the frontal cortex but not the hippocampus following the final dose in survivor mice. (A) Hippocampus, (B) Frontal cortex. Unpaired parametric two-tailed t tests were used for statistical analyses. Nonparametric Mann-Whitney U tests were used when the assumptions of normality and equal variances were not met. *p<0.05 [file Image_2.tiff]

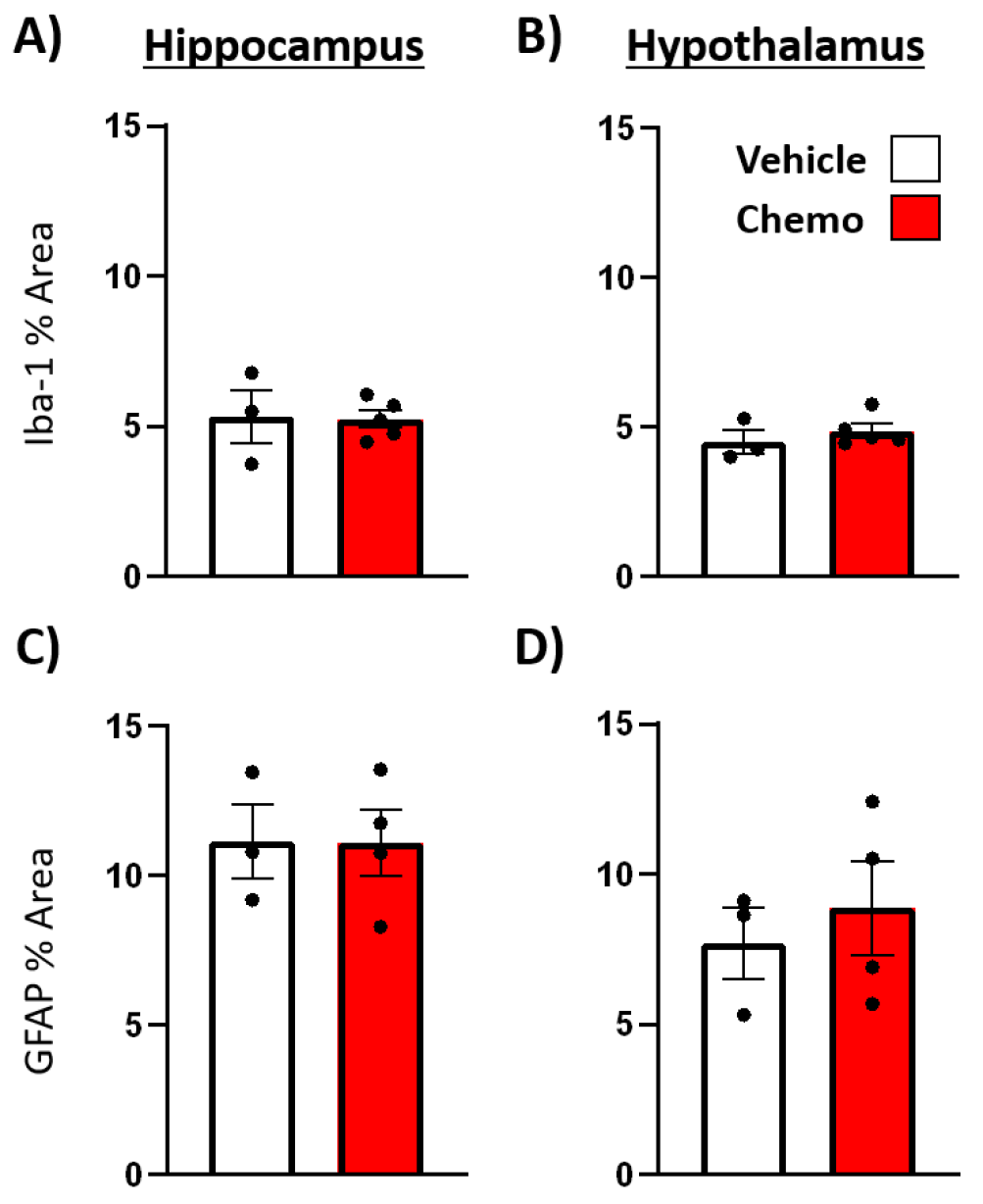

Supplement: Supplementary Figure 3 — Paclitaxel chemotherapy does not alter Iba-1 or GFAP percent area in the hippocampus or hypothalamus following the final dose in survivor mice. (A) Hippocampal Iba-1% area, (B) Hypothalamic Iba-1% area, (C) Hippocampal GFAP % area, (D) Hypothalamic GFAP % area. Paclitaxel n=4, vehicle n=3. Unpaired parametric two-tailed t tests were used for statistical analyses. Nonparametric Mann-Whitney U tests were used when the assumptions of normality and equal variances were not met. *p<0.05. [file Image_3.tiff]

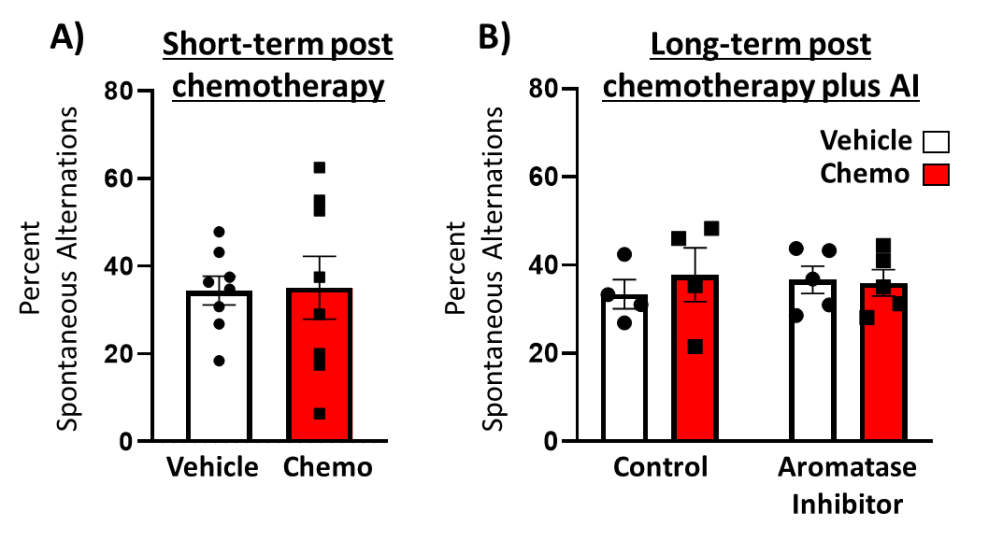

Supplement: Supplementary Figure 4 — Neither paclitaxel chemotherapy nor aromatase inhibitor treatment affected percent spontaneous alternations. (A) Percent spontaneous alternations in the spontaneous alternations test following the final dose of paclitaxel (n=8/group). (B) Percent spontaneous alternations in the spontaneous alternations test following aromatase inhibitor treatment. Aromatase inhibitor n=5, control n=4. Unpaired parametric two-tailed t tests were used for statistical analyses. Nonparametric Mann-Whitney U tests were used when the assumptions of normality and equal variances were not met. *p<0.05. [file Image_4.tiff]
